# Recurrence after radical and partial nephrectomy in high complex renal tumor using propensity score matched analysis

**DOI:** 10.1038/s41598-021-82700-8

**Published:** 2021-02-03

**Authors:** Hwanik Kim, Jung Kwon Kim, Changhee Ye, Joon Hyeok Choi, Hakmin Lee, Jong Jin Oh, Sangchul Lee, Sung Kyu Hong, Seok-Soo Byun

**Affiliations:** 1 Department of Urology, Seoul National University Bundang Hospital, 82 Gumi-Ro, 173 Beon-gil, Bundang-gu, Seongnam-si, Gyeonggi-do 13620 South Korea; 2 Department of Urology, Seoul National University College of Medicine, Seoul, South Korea; 3 Biochemistry, College of Arts and Sciences, Boston College, Newton, MA USA

**Keywords:** Urological cancer, Renal cancer, Cancer, Urology, Oncology, Surgical oncology

## Abstract

We evaluated the recurrence after radical and partial nephrectomy in patients with RENAL nephrometry score [RENAL] ≥ 10. A total of 474 patients (radical nephrectomy [RN, n = 236] & partial nephrectomy [PN, n = 238]) in a single tertiary referral institution from December 2003 to December 2019 were assessed. Functional outcomes, defined as estimated glomerular filtration rate changes, relapse pattern, recurrence-free survival (RFS), cancer-specific survival (CSS), and overall survival (OS) were evaluated using propensity score-matched analysis. The predictors of recurrence and survival were assessed by Cox-regression analysis. 44 patients in the RN group and 88 in the PN group were included without significant differences in preoperative clinical factors after matching. The PN patients achieved significantly higher renal function preservation rates (p < 0.001). There were five recurrences in RN and six in PN. The PN patients revealed 5-year RFS rate (86.8%), 5-year CSS rate (98.5%), and 5-year OS rate (98.5%) comparable to the RN patients (RFS: 88.7% [p = 0.780], CSS: 96.7% [p = 0.375], and OS: 94.3% [p = 0.248]). Patients with a body mass index (BMI) ≥ 23 had lower 5-year RFS rates (85.5%) and OS rates (95.6%) than those with BMI < 23 (RFS: 90.0% [p = 0.195], OS: 100% [p = 0.117]) without significance. The significant predictor of recurrence was the pathologic T stage (hazard ratio [HR] 3.99, 95% confidence [CI] 1.10–14.50, p = 0.036). The significant predictor of death was the R domain of the RENAL (HR 3.80, 95% CI 1.03–14.11, p = 0.046). PN, if technically feasible, could be considered to preserve renal function in patients with RENAL ≥ 10. Nonetheless, PN needs to be implemented with caution in some patients due to the higher potentiality for recurrence and poor survival.

## Introduction

In the localized renal cell carcinoma (RCC), a partial nephrectomy (PN) or radical nephrectomy (RN), by tumor characteristics, is the treatment of choice for surgical candidates^1^. PN is becoming the standard management of clinical T1 tumors^2^ resulting in equivalent oncological outcomes as those of RN, functional preservation, and favorable survival benefit reported from several national database studies and meta-analyses^3,4^.

Nevertheless, 20–40% of the patients treated for the localized case were reported to have recurrences^5^. In a multicenter study, Shah et al. observed that the disease recurred in 5.6% of the patients treated with PN for clinically localized RCC^6^. With the number of PN increasing in the last few years, growing attempts have been made to implement PN even for high complex renal tumors described by RENAL nephrometry score (RENAL), developed as a useful evaluation tool for predicting operative complexity posed by warm ischemic time (WIT) or postoperative complications^7^. Despite technological advances and the adoption of robotic surgical systems, few data on PN for high complex renal masses leave unmet need challenges. Furthermore, there is limited evidence on the recurrence of RCC in patients with high complex renal masses.

In this study, we aimed to investigate the predictors and patterns of RCC recurrence in patients with high complex tumors diagnosed as RCC and treated with PN or RN from a single center and assess the impact on recurrence-free survival (RFS), cancer-specific survival (CSS), and overall survival (OS).

## Methods

### Patient population

The Institutional Review Board of Seoul National University Bundang Hospital approved the current study (approval number: B-2007-625-102). A written informed patient consent was waived by the Seoul National University Bundang Hospital Institutional Review Board due to the retrospective nature of study. Personal identifiers were completely deleted such that data were analyzed anonymously. We reviewed our prospectively maintained institutional database of 3013 patients who underwent RN or PN between December 2003 and December 2019 at a single tertiary referral center. All methods were conducted in accordance with relevant guidelines and regulations (the ethical standards of the 1964 Declaration of Helsinki and its later amendments or comparable ethical standards).

The complexity of surgery was defined by RENAL. Renal masses with an RENAL range of 4–6, 7–9, and 10–12 indicated low, moderate, and high complex lesions, respectively, as described by Kutikov and Uzzo^7^. We strictly defined local recurrence as (1) the detection of a new enhancing lesion in the surgical bed of the original nephrectomy site(s) or regional lymph nodes (LN), which was identified by the urological oncologist or radiologist on follow-up imaging or (2) the detection of a new enhancing lesion in the same region of the ipsilateral kidney as the original PN site (e.g., the PN site and the recurrence site were both in the lower pole)^6^. Systemic recurrence was defined as tumor development at systemic distant sites or in non-regional retroperitoneal LNs^8^.

After excluding 2569 patients with low (n = 742), moderate (n = 1283) complex, or unknown (n = 544) RCC complexity, 153 were initially diagnosed with metastatic RCC with high complex renal masses (44 cN1 cases, 82 cM1 cases, and 27 cN1M1 cases) and 291 with nonmalignant histology (110 angiomyolipomas, 60 oncocytomas, and 121 other benign cysts). Recurrence was noted in 61 of 474 patients (12.9%) with high complex renal masses who underwent RN (n = 236) or PN (n = 238).

All specimens were analyzed by dedicated urological pathologists. A positive surgical margin (PSM) was defined as the extension of the tumor to the inked surface of the resected specimen on the final pathology evaluation.

### Follow-up protocol

According to the standardized institutional postoperative protocol, the patients were generally followed-up after surgery at least every 6 months in the first year, annually during the next 4 years, and every 2 years thereafter. The follow-up protocols consisted of computed tomography or magnetic resonance imaging studies and chest radiography. Renal functional outcomes defined as estimated glomerular filtration rate (eGFR) changes were followed.

RFS was defined as the interval between the date of surgery and the time of the first tumor recurrence. The cause of death was determined by the responsible physicians and death certificates. CSS was calculated from the date of surgery to the date of the last follow-up or death related to renal cell carcinoma. OS was calculated from the date of surgery to the date of the last follow-up or death due to all causes^9^.

### Statistical analyses

The clinicopathological characteristics were compared between the patients who underwent RN and PN using the chi-squared test for categorical variables and the independent t test or Mann–Whitney U test for continuous variables. Kaplan–Meier survival analysis was used to calculate the survival estimates for RFS and OS. Further, the log-rank test was used to conduct comparisons between the groups. Univariate and multivariate Cox proportional hazard regression analyses were performed to evaluate the significant variables associated with the survival outcomes^9^. We conducted an additional analysis to determine if the body mass index (BMI) affected RCC recurrence or survival. The univariate results were used to determine the candidate variables for the final multivariate model in a backward model selection process. In all variables remaining in the final multivariate analysis, the p value was set to 0.05. To provide a further balance between radical and partial nephrectomies, we performed propensity score-matching (PSM). For a binary treatment indicator of the type of surgery (RN vs. PN), PSM was performed using age, sex, BMI, diabetes mellitus (DM) status, hypertension (HTN) status, Eastern Cooperative Oncology Group Performance Status (ECOG PS), Modification of Diet in Renal Disease-GFR (MDRD-GFR), tumor size, clinical T stage, operation technique, and RENAL. Matching variables were selected to balance the variables most likely influencing operative bias. Matching was performed using a 1:2 ratio between the RN and PN groups with a nearest neighbor-matching algorithm^10^. PSM was performed using the MatchIt extension package in R software (Vienna, Austria). All data were analyzed with SPSS version 22, and all tests were 2-sided with a p value of 0.05 considered statistically significant (IBM SPSS Statistics, IBM Corp., Armonk, NY, USA).

## Results

### Patient demographics

Table 1 shows the patient baseline characteristics. Before the propensity score matching, we detected significant differences between the RN group and the PN group in terms of age, BMI, gender, HTN, chronic kidney disease (CKD), serum creatinine level, MDRD-GFR, tumor size, clinical T stage, RENAL, operation technique, and pathological classification. These preoperative clinical factor differences were eliminated by PSM. After PSM, the RN group and the PN group contained 44 and 88 patients, respectively. The median follow-up duration was 37.5 months in both groups (interquartile range (IQR) 12–60 months). There were no significant differences in the pathological results between the groups.

**Table 1:** Baseline characteristics of the patients.

	Prepropensity cohort			Postpropensity cohort		
	RN (n = 236)	PN (n = 238)	p value	RN (n = 44)	PN (n = 88)	p value
Age	57.5 ± 13.4	54.1 ± 12.8	0.005	55.3 ± 11.6	55.5 ± 13.0	0.937
BMI	24.3 ± 3.3	25.0 ± 3.4	0.033	25.3 ± 3.7	25.1 ± 3.4	0.838
**Gender**						
Male/female	149 (63.1%)/87 (36.9%)	175 (73.5%)/63 (26.5%)	0.015	31 (70.5%)/13 (29.5%)	67 (76.1%)/21 (23.9%)	0.482
DM	32 (13.6%)	31 (13.0%)	0.864	7 (15.9%)	14 (15.9%)	1.000
HTN	116 (49.2°%)	92 (38.7%)	0.021	21 (47.7%)	41 (46.6%)	0.902
CKD	8 (3.4°%)	1 (0.4%)	0.020	0	0	1.000
**ECOG PS**			0.104			0.455
0	192 (81.4%)	202 (84.9%)		38 (86.4%)	72 (81.8%)	
> 1	44 (18.6%)	36 (15.1%)		6 (13.6%)	16 (18.2%)	
Serum creatinine	1.1 ± 0.9	0.9 ± 0.2	0.001	0.9 ± 0.2	0.9 ± 0.2	0.543
MDRD GFR	78.6 ± 25.8	89.7 ± 21.8	< 0.001	90.0 ± 27.1	88.1 ± 23.3	0.687
Tumor size (mm)	79.3 ± 67.5	35.9 ± 16.2	< 0.001	47.2 ± 17.4	46.0 ± 16.5	0.693
**Clinical T stage**			< 0.001			0.781
1a/1b	34 (14.4%)/63 (26.7%)	148 (62.2%)/85 (35.7%)		19 (43.2%)/21 (47.7%)	41 (46.6%)/43 (48.9%)	
2/3	87 (36.9%)/52 (22.0%)	3 (1.3%)/2 (0.8%)		3 (6.8%)/1 (2.3%)	3 (3.4%)/1 (1.1%)	
**RENAL nephrometry score**	10.4 ± 0.6	10.2 ± 0.4	< 0.001	10.1 ± 0.3	10.2 ± 0.4	0.748
R	2.3 ± 0.8	1.7 ± 0.7	< 0.001	1.6 ± 0.7	1.7 ± 0.6	0.851
E	2.2 ± 0.7	2.6 ± 0.6	< 0.001	2.6 ± 0.6	2.6 ± 0.5	0.589
N	3.0 ± 0.2	3.0 ± 0.1	0.312	3.0 ± 0.0	3.0 ± 0.0	1.000
A (a/p/x)	39.8%/44.9%/15.3%	39.1%/31.1%/29.8%	< 0.001	45.5%/29.5%/25.0%	42.0%/36.4%/21.6%	0.730
L	2.9 ± 0.3	2.9 ± 0.3	0.851	3.0 ± 0.2	2.9 ± 0.3	0.214
**Technique**			< 0.001			0.877
Laparoscopic/open	54 (22.9%)/101 (42.8%)	4 (1.7%)/61 (25.6%)		1 (2.3%)/14 (31.8%)	3 (3.4%)/25 (28.4%)	
Robot	81 (34.3%)	173 (72.7%)		29 (65.9%)	60 (68.2%)	
**Pathological T stage**			< 0.001			0.335
1a/1b	31 (13.1%)/56 (23.7%)	156 (65.5%)/66 (27.7%)		15 (34.1%)/19 (43.2%)	39 (44.3%)/38 (43.2%)	
2	58 (24.5%)	4 (1.7%)		3 (6.8%)	4 (4.5%)	
3/4	87 (36.9%)/4 (1.6%)	12 (5.0%)/0		7 (15.9%)/0	7 (8.0%)/0	0.478
pN1/pM1	1/1	0/0	< 0.001/1.000	0/0	0/0	1.000
**Furhman’s grade**			< 0.001			0.856
I/II	1 (0.4%)/52 (22.0%)	2 (0.8%)/83 (35.0%)		0/11 (25.0%)	1 (1.1%)/23 (26.1%)	
III/IV	137 (58.1%)/46 (19.5%)	133 (56.1%)/19 (8.0%)		26 (59.1%)/7 (15.9%)	53 (60.2%)/11 (12.5%)	

*RN* radical nephrectomy, *PN* partial nephrectomy, *BMI* body mass index, *DM* diabetes mellitus, *HTN* hypertension, *ECOG* Eastern cooperative oncology group performance status, *MDRD* modification of diet in renal disease, *GFR* estimated glomerular filtration rate, *R* radius (maximal diameter), *E* exophytic/endophytic, *N* nearness to collecting duct system/renal sinus, *A* anterior/posterior location, *L* location relative to the polar lines.

### Effect of operation type (RN vs. PN) on renal function

**Figure 1:**
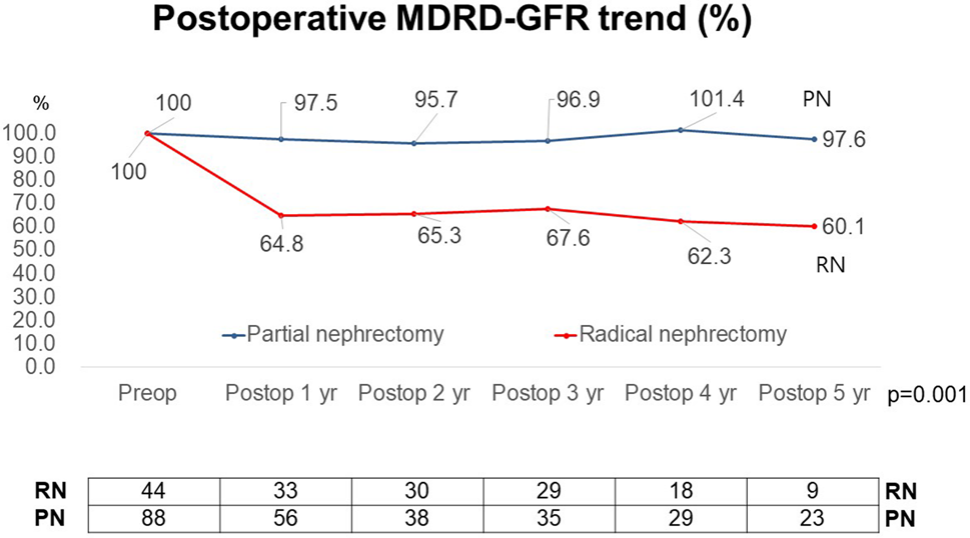
Postoperative renal function trends in glomerular filtration rate (GFR) preservation rates after radical nephrectomy and partial nephrectomy between the groups.

There were significant differences in renal function between the two groups throughout the follow-up period (p = 0.001) (Fig. 1). At the follow-up 1 year after surgery, 90% eGFR preservation rates were found in 6.1% of the patients in the RN group and 70.2% of the patients in PN group (p < 0.001). Moreover, de novo CKD stage III or higher incidence rates at postoperative 1 year were seen in 41.1% of the patients in the RN group and 3.6% in the patients in the PN group (p < 0.001).

### Effect of operation type and BMI on recurrence and overall survival

**Figure 2:**
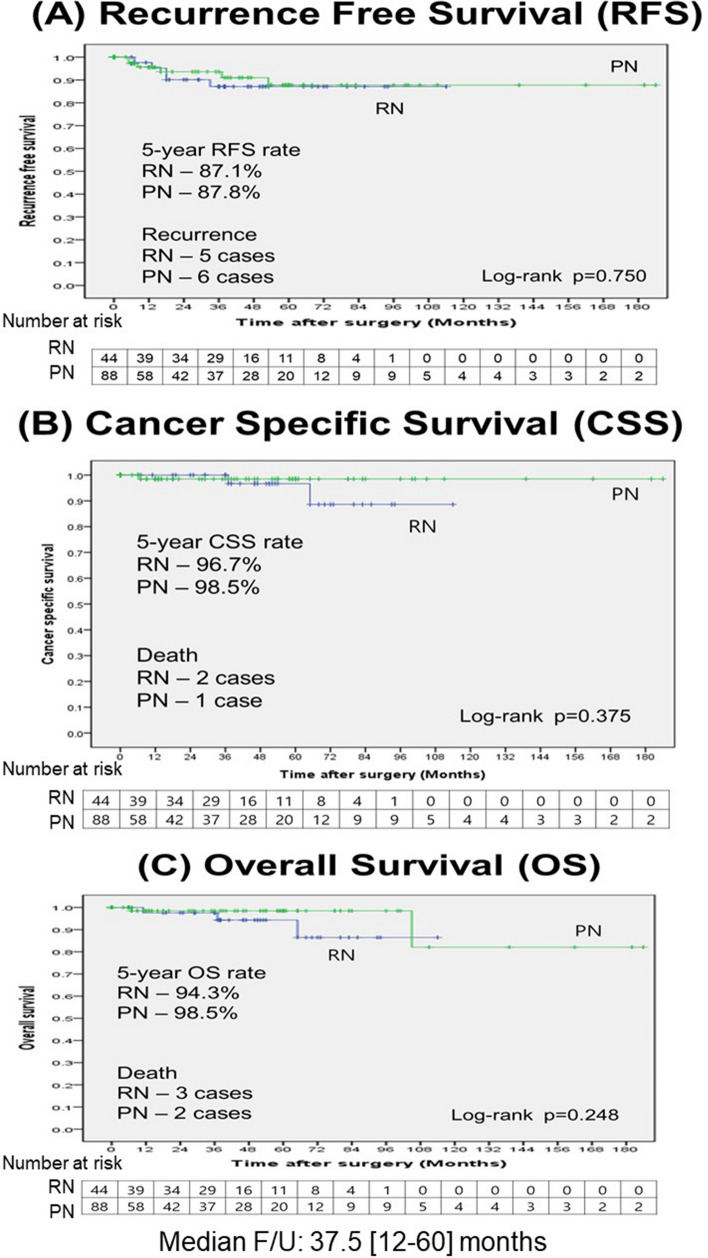
Kaplan–Meier curves for recurrence-free survival (RFS), cancer-specific survival (CSS), and overall survival (OS) between the groups. (**A**) RFS, (**B**) CSS, and (**C**) OS.

Among the patients in the post-propensity cohort, 11 patients (8.34%) were found to fit the criteria for recurrence. There were five systemic recurrences in the RN patients and six in the PN patients. There were no local recurrences. Five patients died during follow-up, which included three cancer-specific deaths in the RN group, and one cancer-specific death in the PN group. The PN patients had 5-year RFS rate (86.8%), 5-year CSS rate (98.5%), and 5-year OS rate (98.5%) comparable to the RN patients (RFS rate, 88.7% [p = 0.780]; CSS rate, 96.7% [p = 0.375]; OS rate, 94.3% [p = 0.248]). There were no differences in any type of survival between the two groups after PSM (Fig. 2).

In the BMI, before PSM, the 5-year RFS rate was significantly higher in patients with BMI ≥ 23 (88.8% vs. 73.4%, p = 0.001). After PSM, the patients with BMI ≥ 23 had lower 5-year RFS rates (85.5%) and OS rates (95.6%) than the patients with BMI < 23 (RFS, 90.0% (p = 0.195); OS, 100% (p = 0.117)) without significance (Supplementary Figure 1).

The significant predictor of recurrence was the pathologic T stage (hazard ratio [HR] = 3.99, 95% confidence [CI]: 1.10–14.50, p = 0.036) (Table 2). The significant predictor of death was the R portion of the RENAL (HR 3.80, CI 1.03–14.11, p = 0.046) (Table 3). The patterns and management of recurrence between the groups are detailed in Supplementary Tables 1 and 2.

**Table 2:** Univariate and multivariate analysis for recurrence after nephrectomy.

	Univariate analysis			Multivariate analysis		
	HR	CI	p value	HR	CI	p value
Age	1.01	0.96–1.06	0.689			
**Asian BMI standard**						
BMI < 23	Ref		0.465			
23 ≤ BMI < 30	3.48	0.44–27.48	0.237			
BMI ≥ 30	4.5	0.28–72.21	0.288			
BMI ≥ 23	3.56	0.46–27.83	0.226			
**Gender**						
Male—Ref	3.17	0.97–10.37	0.057			
DM	0.51	0.07–3.978	0.519			
HTN	1.3	0.40–4.25	0.669			
ECOG	1.89	0.75–4.76	0.177			
Serum Cr	0.78	0.07–8.88	0.844			
GFR	0.99	0.96–1.01	0.373			
Tumor size	1.05	1.01–1.10	0.012	1.02	0.98–1.07	0.307
**Op technique**						
PN	0.82	0.25–2.71	0.751			
Robot	Ref		1.000			
Laparoscopy	0	0	0.984			
Open	1.02	0.30–3.40	0.981			
**RENAL score**	1.52	0.47–4.94	0.487			
R	2.55	1.16–5.64	0.020	1.08	0.29–4.05	0.117
E	0.37	0.17–0.82	0.015	0.47	0.11–1.97	0.302
N	0	0	1.000			
A						
a	Ref		0.480			
p	0	0.00–1078	0.959			
x	0.39	0.08–1.80	0.226			
L	1.09	0.14–8.55	0.932			
**Pathologic T stage**						
pT1	Ref		0.003	Ref		0.036
pT2-pT3	6.10	1.86–20.02		3.99	1.10–14.50	

*BMI* body mass index, *DM* diabetes mellitus, *HTN* hypertension, *ECOG* Eastern cooperative oncology group performance status, *GFR* estimated glomerular filtration rate, *Op* operation, *PN* partial nephrectomy, *RENAL score* RENAL nephrometry score, *R* radius (maximal diameter), *E* exophytic/endophytic, *N* nearness to collecting duct system/renal sinus, *A* anterior/posterior location, *L* location relative to the polar lines, *HR* hazard ratio, *CI* confidence interval, *Ref* reference.

**Table 3:** Univariate and multivariate analysis for overall survival after nephrectomy.

	Univariate analysis			Multivariate analysis		
	HR	CI	p value	HR	CI	p value
Age	1.01	0.94–1.08	0.887			
BMI ≥ 23	44.06	0.01–151,827.2	0.362			
**Gender**						
Male—Ref	2.74	0.39–19.50	0.314			
DM	3.54	0.59–21.22	0.166			
HTN	4.41	0.49–39.56	0.185			
ECOG	0.06	0–2103.6	0.599			
Serum Cr	0.79	0.01–48.91	0.912			
GFR	0.99	0.95–1.03	0.991			
Tumor size	1.03	0.97–1.09	0.378			
**Op technique**						
PN	2.88	0.45–18.61	0.266			
Robot	Ref		0.898			
Laparoscopy	0	0	0.990			
Open	1.54	0.25–9.62	0.644			
**RENAL score**	1.17	0.11–8.50	0.880			
R	3.8	1.03–14.11	0.046	3.8	1.03–14.11	0.046
E	0.32	0.09–1.08	0.066	0.75	0.15–3.78	0.725
N	0	0	1.000			
A						
a	Ref		0.868			
p	0.54	0.06–5.42	0.603			
x	0.75	0.08–7.41	0.806			
L	0.22	0.02–2.40	0.212			
**Pathologic T stage**						
pT1	Ref		0.141			
pT2-pT3	4.37	0.61–31.16				

*BMI* body mass index, *DM* diabetes mellitus, *HTN* hypertension, *ECOG* Eastern cooperative oncology group performance status, *GFR* estimated glomerular filtration rate, *Op* operation, *PN* partial nephrectomy, *RENAL score* RENAL nephrometry score, *R* radius (maximal diameter), *E* exophytic/endophytic, *N* nearness to collecting duct system/renal sinus, *A* anterior/posterior location, *L* location relative to the polar lines, *HR* hazard ratio, *CI* confidence interval, *Ref* reference.

## Discussion

Several large retrospective studies have been conducted on the recurrence rates after surgical treatment of RCC. Notably, the majority of the data included patients with nephrectomy in the 1980s–1990s. All studies derived from institutional cohorts without hospital-based registries or population-based cohorts. Overall, the 5-year RFS rates were from 41.9 to 97.8%. However, the cohorts were diversely distributed in aspects of the disease stages and surgical methods (PN vs. RN and laparoscopic vs. open techniques). One contemporary cohort was comprised of 1541 patients who underwent PN for clinical T1a and T1b tumors from 1999 to 2008^11^. Distant metastases were found in 59 patients (4.9%) after nephrectomy. The 5-year RFS rates were between 97.1 and 97.8% for clinical T1a and between 92.7 and 93.1% for clinical T1b tumors. Though no studies have directly compared the recurrence rates in previous versus more contemporary cohorts for localized RCC, the 5-year RFS is likely to be more than 90% in T1 patients following surgery^5^. Unlike those results, our post-propensity cohort revealed a relatively lower 5-year RFS (RN: 88.7% vs. PN: 86.8%), but the results should be interpreted with caution as there were fewer patients at risk in each group at serial yearly follow-ups.

Local recurrence in the renal fossa after RN has been studied well. Itano et al. reported a 1.8% of local recurrence rate after RN^12^. The 5-year CSS was poor at 28% in those patients but expanded with surgical resection of the recurrence. Thomas et al. reported that the pathological nodal stage at the original nephrectomy and the maximal diameter of the retroperitoneal recurrence were independent risk factors for CSS^13^. Margulis et al. found the same recurrence rate (1.8%) in 2009 and correlated specific clinical factors with worse CSS^14,15^. Compared to these results, the pre-propensity patients in the RN group in our study had no isolated local recurrences but eight (3.4%) had combined local and systemic recurrences. Seven had renal fossa recurrences and one had regional LN recurrence, all followed by concurrent or subsequent systemic metastasis. However, the post-propensity cohort showed only systemic recurrences.

As mentioned, few studies have been conducted on local recurrence after nephrectomy for high complex renal masses. All were limited by the small number of patients in the cohorts and provided broad and variable definitions of local recurrence. From an observational study of 360 sporadic and nonfamilial patients with T1 tumors who received laparoscopic partial nephrectomy, Kreshover et al. reported that 1.4% of the patients experienced local recurrence in the retroperitoneum or the operated kidney^16^. In a contemporary retrospective study by Thompson et al., the definition of local recurrence was a mass in the operated kidney. They found a 3.4% local recurrence rate in cT1a tumors (36 of 1057) and a 6.4% for cT1b tumors (21 of 326) after open PN^17^. The another contemporary review of 279 patients with a mean follow-up of 25 months (IQR 7–43) by Garisto et al. reported that 4.3% of the total patients had recurrences, 4.43% and 3.95% in the robot-assisted PN and the open PN groups, respectively (p = 0.6). They also observed that both the open and robotic approach led to a significant decrease in postoperative eGFR^18^. Our cohort with propensity score matching showed an RFS rate similar to a large sample-sized study^18^ of a population with highly complex renal masses but had longer follow-up and better preserved renal function. The RFS rates of patients with high complex renal masses did not differ significantly by treatment approach in the current study.

The association between BMI and mortality has been observed in patients with RCC across several cohorts. It is well known that obese patients with localized clear cell RCC who are treated with nephrectomy survive longer than those with normal weight according to the World Health Organization (WHO) categorization (BMI 18.5–24.9 kg/m^2^), a phenomenon known as the obesity paradox^19^. A meta-analysis of patients with RCC who underwent nephrectomies showed higher OS in overweight or obese versus normal-weight patients (pooled HR 0.57, CI 0.43–0.76)^20^ A recent study reported differences in the tumor microenvironment in obese patients relative to normal patients^19^. Although we evaluated the effect of BMI on tumor recurrence or survival, we only found a significant RFS difference in the pre-propensity cohort. While this finding might be attributed to significantly different demographics between the groups, our unexpected findings are supported by other studies and we confirmed that the obesity paradox also applied to our cohort^21–23^.

The current study had some limitations, including the retrospective nature and single-center design with a small comparison group. In addition, the results may not be replicated in patients with a solitary kidney or metastatic RCC, as we only included patients with non-metastatic bilateral kidneys. Despite these limitations, to our knowledge, this was the first study identifying preoperative clinical factors associated with the recurrence and survival of RCC patients with high complex renal masses, overcoming the relatively low number of patients.

## Conclusions

In patients with RENAL ≥ 10, PN should be performed to preserve renal function if technically feasible. We can confidently propose broadening the PN indication to include high complex tumors, regardless of the surgical difficulty. Nevertheless, PN should be done with caution in some cases due to the higher potential for recurrence and poor survival. Longer follow-up studies with larger cohorts and randomized controlled trials are expected to verify these findings.

## Supplementary Information

Supplementary Information.
