# Supplementary material for: Recurrence after radical and partial nephrectomy in high complex renal tumor using propensity score matched analysis
Source: Sci Rep. 2021 Feb 3;11:2919. doi: 10.1038/s41598-021-82700-8 (PMC7859240; doi:10.1038/s41598-021-82700-8)

Recurrence after radical and partial nephrectomy in high complex renal tumor using propensity score matched analysis

Hwanik Kim^1^, Jung Kwon Kim^1^, Changhee Ye^1^, Joon Hyeok Choi^3^, Hakmin Lee^1^, Jong Jin Oh^1^, Sangchul Lee^1^, Sung Kyu Hong^1,2^, Seok-Soo Byun ^1,2^

^1^ Department of Urology, Seoul National University Bundang Hospital, South Korea

^2^ Department of Urology, Seoul National University College of Medicine, Seoul, South Korea

^3^ **Biochemistry, College of Arts and Sciences, Boston College**, United States

Corresponding author : Seok-Soo Byun ssbyun@snubh.org

Professor, Department of Urology, Seoul National University College of Medicine, Seoul National University Bundang Hospital, Seongnam, Korea

166 Gumi-Ro, Bundang-gu, Seongnam-si, Gyeonggi-do, 463-707, Korea

Tel: 82-31-787-7342, Fax: 82-31-787-4057, E-mail: ssbyun@snubh.org

Supplementary Table 1. Recurrence patterns in the post-propensity patients

| Variable | RN (n=44) | PN (n=88) | p-value |
| --- | --- | --- | --- |
| Recurrence rate | 5 (11.4%) | 6 (6.8%) | 0.564 |
| Recurrence type |  |  |  |
| Local | 0 | 0 | 0.251 |
| Systemic | 4 | 6 |  |
| Local + systemic | 1 | 0 |  |
| Recurrence site |  |  | 0.491 |
| Mass base | 1 | 0 |  |
| Lung | 3 | 5 |  |
| Adrenal gland | 1 | 1 |  |
| Multiple sites | 1 | 0 |  |

Supplementary Table 2. Recurrence management of the post-propensity patients

|  | RN (n=5) | PN (n=6) |
| --- | --- | --- |
| Metastasectomy | 2 | 3 |
| Radiation therapy | 1 | 0 |
| Target therapy | 3 | 1 |
| Lost to follow-up | 0 | 1 |

Supplementary Figure 1. Curves for recurrence-free survival (RFS) according to BMI 23. (A) RFS-before PSM and (B) RFS-after PSM.


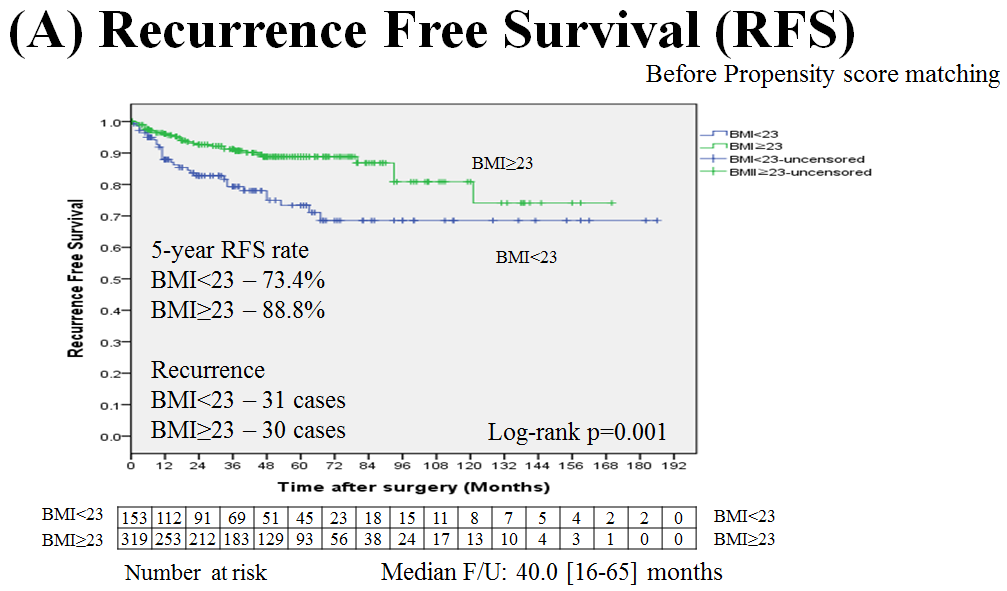


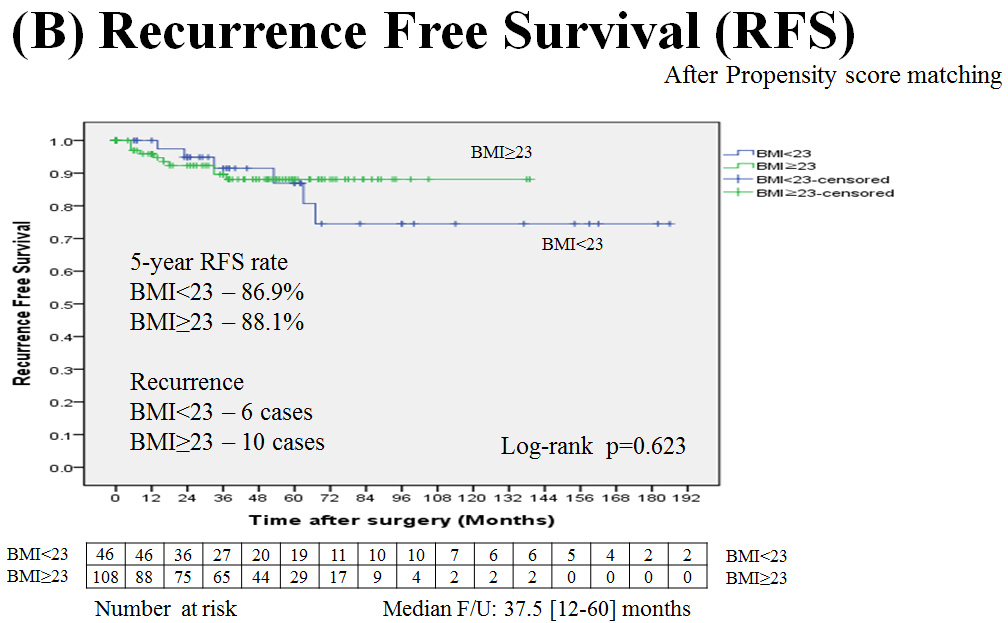

Supplement: Supplementary file 1 — Supplementary Information. [file 41598_2021_82700_MOESM1_ESM.docx]
